# Cerebrospinal fluid biomarkers in patients with plasma HIV RNA below 20 copies/mL

**DOI:** 10.7448/IAS.17.4.19719

**Published:** 2014-11-02

**Authors:** Andrea Calcagno, Cristiana Atzori, Alessandra Romito, Sara Ecclesia, Daniele Imperiale, Sabrina Audagnotto, Maria Chiara Alberione, Alice Trentalange, Giovanni Di Perri, Stefano Bonora

**Affiliations:** 1 Department of Medical Sciences, University of Torino, Torino, Italy; 2 Unit of Neurology, Ospedale Maria Vittoria, ASLTO2, Torino, Italy; 3 Laboratory of Immunology, Ospedale Amedeo di Savoia, ASLTO2, Torino, Italy

## Abstract

**Introduction:**

Low level HIV-1 CSF replication (CsfLLV) is often found even in patients with controlled plasma viraemia. The clinical consequences of such finding are uncertain; however, both symptomatic neurological disturbances and neurocognitive disorders may arise in the context of CSF-escape. Two reports suggested that low level replication in the CSF may be associated with increased CSF neopterine although the impact on other markers of neuroinflammation/damage is currently unknown.

**Materials and Methods:**

Patients with neurocognitive disorders, new neurological symptoms or followed in longitudinal studies were included provided that they were on HAART, with last available viral load below 20 copies/mL and without central nervous system (CNS)-involving infections/neoplasms. After brain Magnetic Resonance (MR) CSF HIV RNA (CAP/CTM HIV-1 v2.0) and biomarkers [total tau (t-tau), phosphorylated tau (p-tau), 1–42 Beta amyloid (Beta42), neopterine and S100beta] were measured through validated methods. Data are presented as medians (IQR); non-parametric tests are used for all analysis.

**Results:**

70 patients [66.7% male, median age 47.8 years (40–56), median BMI 22.2 kg/m2 (20–24)] were enrolled. Current and nadir CD4+ cell count were 379 (219–656) and 116 cell/mm3 (46–225); HIV RNA was undetectable since 19.7 months (9–53). CSF HIV RNA was undetectable in 24 (34.3%), below 20 copies/mL in 26 (37.1%), above 20 copies/mL in 25 patients [35.7%, median 69 copies/mL (41–134]). Median (IQR) CSF biomarkers values were as follows: t-tau 109 pg/mL (<75–161), p-tau 31.6 pg/mL (23.4–35.4), Beta42 818 pg/mL (623–973), neopterine 0.58 ng/mL (0.45–0.87) and S100beta 149 pg/mL (110–186). Patients with CsfLLV did not show significant differences as for demographic, therapeutic, virological, radiological variables. t-tau (134 vs 92.6, p=0.05) and Beta42 (953 vs 675, p=0.007) were higher in patients with CsfLLV. Neopterine levels were directly associated with p-tau (rho=0.42, p=0.01), with CSF HIV RNA (rho=0.24, p=0.06). and inversely with current CD4 cell count (rho=−0.29, p=03).

**Conclusions:**

In patients with controlled HIV viraemia (below 20 copies/mL), CSF total tau, Beta42 and neopterine were higher in patients with detectable HIV RNA. Prospective and adequately powered studies are warranted for evaluating the clinical significance of compartmental viral replication and immune activation.

